# A Low-Profile Balanced Dielectric Resonator Filtering Power Divider with Isolation

**DOI:** 10.3390/mi16010088

**Published:** 2025-01-14

**Authors:** Rong Cai, Chuan Shao, Kai Xu

**Affiliations:** 1 Information Engineering School, Jiangsu College of Engineering and Technology, Nantong 226007, China; cairong@jcet.edu.cn (R.C.); ch-shao@jcet.edu.cn (C.S.); 2 School of Information Science and Technology, Nantong University, Nantong 226019, China

**Keywords:** balanced, dielectric resonator, filtering power divider, isolation

## Abstract

A balanced dielectric resonator filtering power divider with isolation performance is proposed. By using the coupling of the ${TE}_{111}^{y}$ modes between three rectangle dielectric resonators, combined with balanced feed structures, the differential-mode filtering and power dividing functions, as well as the common-mode suppression were achieved effectively. Additionally, by technically utilizing the hollow structure of the stacked substrates, isolation resistor structures are introduced at the two output ports to improve the isolation level of the power divider. It can solve the problem of traditional metal-cavity dielectric resonator filter power dividers being unable to add isolation structures due to structural reasons. Compared with the reported dielectric resonator filtering power dividers, the proposed one has the characters of a lower profile and high isolation. For demonstration, one dielectric resonator filtering power divider was fabricated and measured at 11.65 GHz with the profile of 0.66 λ_g_ and an isolation higher than 15 dB. The simulation results are in good agreement with the measured results.

## 1. Introduction

With the rapid development of wireless communication, the balanced system has been widely studied due to the advantages of high common-mode suppression. A balanced filter [1,2,3,4,5,6,7] and balanced power divider [8,9,10,11,12,13,14] are two important components of the balanced system, and these two independent devices are usually used in cascade in a traditional RF system. A balanced filtering power divider has both the functions of the filtering, and power dividing has a wide application prospect, which can miniaturize the size of the RF front-end. At the same time, dielectric resonators have become a research hotspot in recent years because of their advantages of high design freedom, high temperature stability, and high power capacity. Therefore, it is of great research value and significance to apply a dielectric resonator to a balanced filtering power divider to form the balanced dielectric resonator filtering power divider.

To our knowledge, most reported balanced power dividers are achieved based on metal resonators. At the beginning, some full-balanced and balanced-to-unbalanced filtering power dividers [15,16,17,18,19] based on half-wavelength microstrip resonators were reported for differential-mode power dividing and common-mode suppression, as well as port isolation. Then, based on the symmetric mode of the patch resonator [20,21,22], some balanced filtering power dividers are proposed for compact size. For the above metal balanced power divider fabricated on PCB (Printed Circuit Board) technology, the isolation between the output ports can be easily obtained by adding the resistors between the microstrip lines on the substrate.

Compared with a balanced metal filtering power divider, there are few reported dielectric resonator filtering power dividers. And parts of them are designed with single-ended ports. The first is a dual-frequency dual-mode dielectric resonator filtering power divider with a flexible output phase difference and power division proposed by Wei Yu [23]. The design is based on the structure of a two-mode resonator loaded with rectangular dielectric blocks with two cut angles in the metal cavity and fed by a probe. Since the external mass factor Qe of the two modes can be controlled independently, the design process of any power distribution ratio is simple and efficient. At the same time, by changing the quadrant where the feed probe is located, the phase difference between the output ports can be adjusted to be in phase or out of phase. The other single-ended dielectric resonator filtering power divider is proposed for dual-band application by using a quad-mode dielectric resonator [24].

In order to further reduce the volume of the filtering power divider and realize the common-mode suppression, Li-Hui Yang proposed a miniaturized balanced filtering power divider based on a single-cavity multi-mode dielectric resonator to achieve an arbitrary power ratio [25]. The design was realized by using two metal cavity loaded cylindrical dielectric resonators combined with balanced probe feeding. By tuning the Qe of the output port, the power ratio can be arbitrarily controlled if the total output Qe is equal to the input Qe. Unfortunately, most of the above designs are concerned about the power distribution ratio and the output phase, and because of the limitation of the metal cavity structure. Therefore, a dielectric resonator filtering power divider with isolation performance is needed to be proposed.

According to the existing problems and challenges of a dielectric resonator filtering power splitter, a balanced filtering power splitter with isolation performance is proposed in this paper. The proposed design firstly realizes the filtering and power division performance through reasonable arrangement and coupling between resonators. Secondly, the isolation resistance structure was added to one layer of the substrate by using the layered structure of the substrate, which solves the problem that the isolation structure cannot be added to the metal cavity structure.

## 2. Proposed Balanced Dielectric Resonator Filtering Power Divider

### 2.1. Configuration

**Figure 1:**
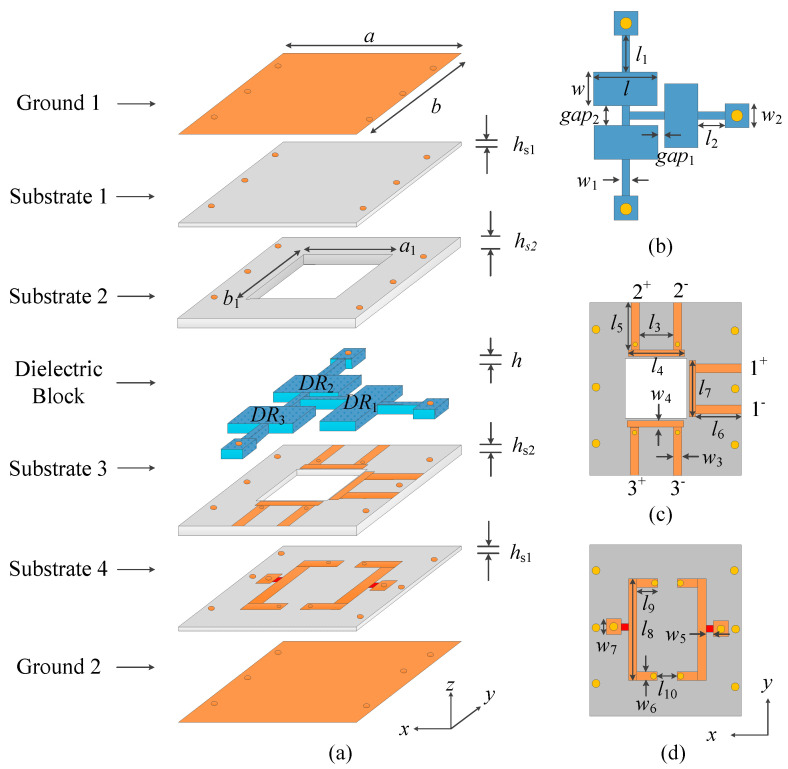
The structure of the proposed filtering power divider. (**a**) Three-dimensional view. (**b**) Dielectric block. (**c**) Substrate 3. (**d**) Substrate 4.

Figure 1 exhibits the configuration of the proposed balanced dielectric resonator filtering power divider. It consists of two ground layers (Ground 1 and Ground 2), four substrate layers (Substrate 1, Substrate 2, Substrate 3 and Substrate 4), and one dielectric layer. Substrates 1–4 are RO4003C substrates with a dielectric constant of 3.55 and a dielectric loss tangent of 0.0027, where Substrate 2 and Substrate 3 are hollowed out in part. And the dielectric layer is the ceramic with the dielectric constant of 9.9 and the dielectric loss tangent of 0.00015. This dielectric layer is mainly composed of three rectangular dielectric blocks (Resonator 1, Resonator 2 and Resonator 3), the connecting strip in the middle, and the fixed strip on both sides. One pair of balanced input ports and two pairs of balanced output ports are printed on Substrate 3. The isolation structure was printed on Substrate 4 and connected to two pairs of output ports on Substrate 3 through two pairs of metal vias on the upper and lower sides. The metal here used were all copper. The simulation software was the 3D electromagnetic simulation software CST 2024 (Computer Simulation Technology).

### 2.2. Analysis of the Dielectric Reaonator

**Figure 2:**
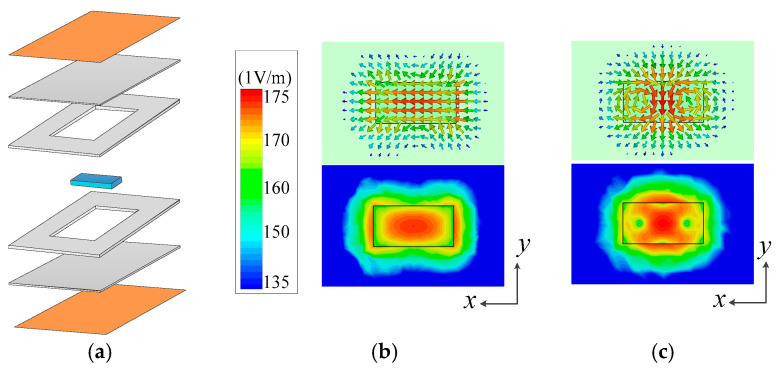
Resonator structure and mode field distribution diagram. (**a**) Resonator structure. (**b**) The magnetic field of the ${TM}_{111}^{y}$ mode. (**c**) The magnetic field of the ${TM}_{111}^{z}$ mode.

Figure 2 shows the three-dimensional structure of the dielectric resonator and the corresponding electric field distribution of the two modes. The resonator structure is shown in Figure 2a, which consists of two ground layers, two whole substrates, two hollow substrates and a rectangular dielectric block. In particular, rectangular hollows were made on the substrate adjacent to the upper and lower layers of the rectangular dielectric block, which can effectively improve the no-load quality factor Qu of the resonator. Figure 2b,c shows the two operating modes in the resonator. The electric field of the ${TE}_{111}^{y}$ mode was concentrated in the center of the rectangular dielectric block, while that of the ${TE}_{211}^{z}$ mode was concentrated in the outer circle of the center, and the electric field of both modes was symmetrically distributed. Since the electric field of the ${TE}_{111}^{y}$ mode was reversed at the long side of the rectangle, it can be excited by a balanced feed. In forming the filtering response, the ${TE}_{111}^{y}$ mode was used as the mode for constructing the differential mode passband, while the ${TE}_{211}^{z}$ mode was used as the mode for constructing the common mode passband.

**Figure 3:**
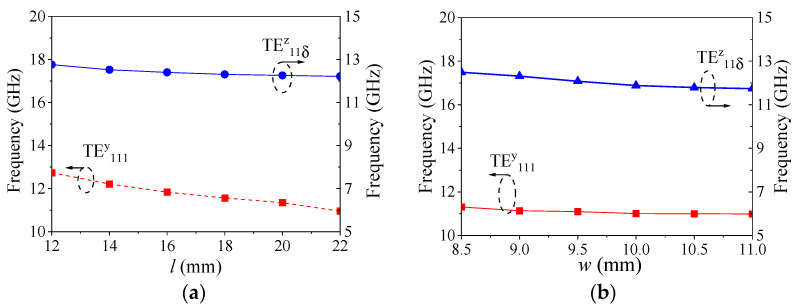
The frequency variation of the mode under different resonator sizes: (**a**) different *l*; (**b**) different *w*.

Figure 3 shows the frequency variation of the two modes under different resonator sizes (length *l* and width *w*). As the length of the resonator increased, the frequencies of both modes decreased, which conforms to the laws of physics, and the frequencies of the two modes were always far apart. Similarly, as the resonator width increased, the frequencies of the two modes also decreased and did not approach each other. That is, the differential mode and common mode did not resonate at the same frequency, which helped achieve good common-mode suppression when the subsequent balanced power divider with filtering responds. According the change rules in Figure 3, the initial values of the resonator can be achieved according to the required frequency. The resonant frequency is controlled by both the length, width, height, and dielectric constant of the resonator. The length or width is just one part of the them; therefore, the frequency changes are weaker.

**Figure 4:**
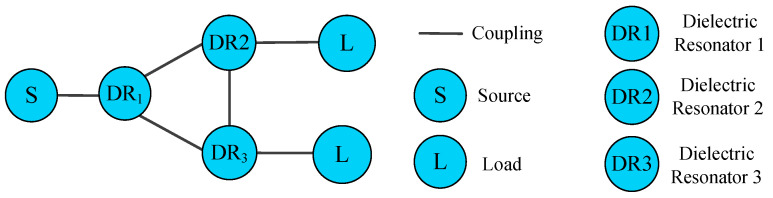
Diagram of the design topology structure.

Figure 4 shows the topology of the dielectric resonator filtering power divider [26], where S and L represent the source and the load, respectively, and DRi is the dielectric resonator. As can be seen, the dielectric resonator is the unit that builds the entire circuit. The whole topology completely shows the transmission process of the signal: first, from the source to the input resonator 1, and then from the input resonator 1 to the two output resonators 2 and 3, and finally from the two output resonators to transmit the signal to the load L. In this process, there will also be a certain coupling between resonators 2 and 3, which will cause the output isolation level of the power splitter to decrease, thus building a 1-to-2 dielectric resonator filtering power divider.

### 2.3. Theoretical Analysis

**Figure 5:**
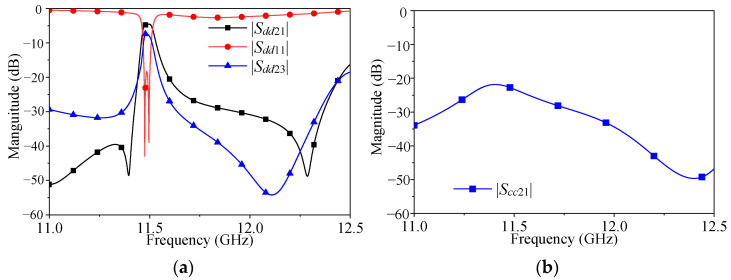
Diagram of the design without the simulation response of the isolated structure: (**a**) differential mode response; (**b**) common-mode response.

Figure 5 shows the simulation results of the filtering power divider without the isolation structure. The detailed dimensions are shown as follows: *a* = 40 mm, *b* = 55.6 mm, *a*_1_ = 22.2 mm, *b*_1_ = 31.4 mm, *l* = 18 mm, *l*_1_ = 5.9 mm, *l*_2_ = 6.2 mm, *l*_3_ = 13 mm, *l*_4_ = 18 mm, *l*_5_ = 8.35 mm, *l*_6_ = 8.63 mm, *l*_7_ = 19 mm, *l*_8_ = 25.1 mm, *l*_9_ = 7 mm, *l*_10_ = 14 mm, *w* = 9 mm, *w*_1_ = 1 mm, *w*_2_ = 3 mm, *w*_3_ = 2.5 mm, *w*_4_ = 0.5 mm, *w*_5_ = 0.6 mm, *w*_6_ = 1.8 mm, *w*_7_ = 1 mm, *gap*_1_ = 2.4 mm, *gap*_2_ = 4.2 mm, *h* = 3.1 mm, *h*_s1_ = 0.203 mm, and *h*_s2_ = 0.813 mm. As shown in Figure 5a, the power divider operated at 11.5 GHz in the K-band with a 3 dB bandwidth of 0.52% for narrow-band applications, and had both one transmission zero on the left and right sides of the passband, which were located at 11.4 GHz and 12.3 GHz, respectively. Therefore, differential-mode filtering and power dividing functions were obtained. However, it can be seen from the figure that the blue line represents the isolation level between the output ports only reached 7.26 dB, which cannot meet the requirement of the power divider. Figure 5b shows the common-mode response of the filtering power divider. Since the differential mode and the common mode did not resonate at the same frequency, the common-mode response was kept below 20 dB within the differential-mode operating frequency band.

**Figure 6:**
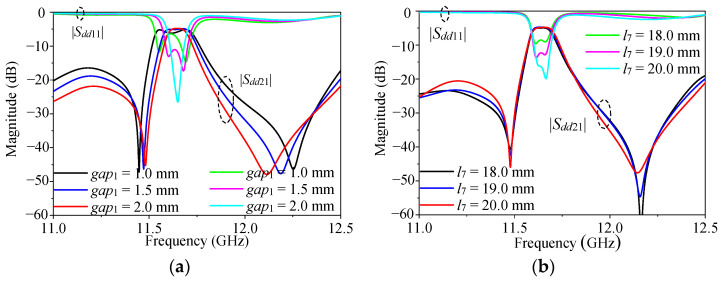
The amplitude response of the design varies with the gap and feed position: (**a**) different *gap*_1_; (**b**) different feed position *l*_7_.

Figure 6 shows how the amplitude response of this design varied with *gap*_1_ and *l*_3_. As can be seen from Figure 6a, the bandwidth of the working frequency band changed with the distance of the coupling distance of the resonator, the coupling distance increased, and the coupling between the resonators became weak, so the bandwidth also became narrow. As can be seen from Figure 6b, with the increase in *l*_7_, the impedance matching became better. This is because the external quality factor Q_e_ is determined by the feed position, and the passband response changes as the feed position changes. According to the required matching and bandwidth, the initial value of the feeding position, as well as the gap, can be achieved.

### 2.4. Improved Isolation Perfermance

As shown in Figure 5 above, the isolation level of this design only reached 7.26 dB, which is far from meeting the performance requirements of the current microwave system for the filtering power divider. Therefore, in order to improve the isolation level, this design used its own layered substrate structure to increase the isolation resistance between the two output ports, and effectively improved the isolation level of the filtering power divider.

**Figure 7:**
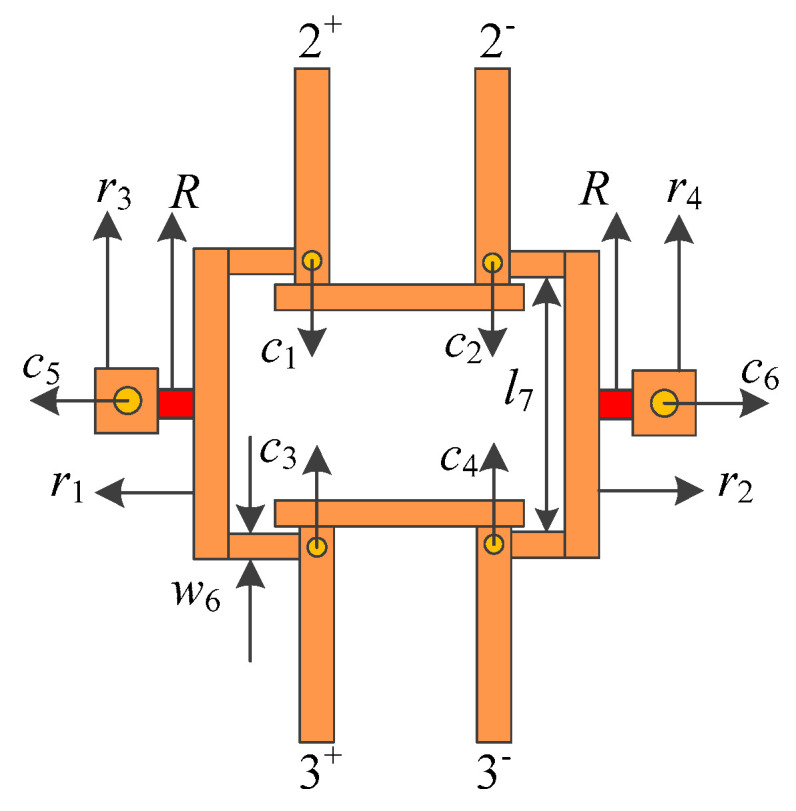
The structure of the isolation circuit.

Figure 7 shows the structure of the isolation circuit. Two pairs of balanced output feeders were printed on Substrate 3. Four metal vias (c_1_, c_2_, c_3_, and c_4_) were introduced into the inner end of each output feeder near the coupled feeder. The two isolation resistors, *R*_1_ and R_2_, were, respectively, connected in the middle of the metal strip r1 and r2, and printed on Substrate 4, and the metal strip was connected with the output feeder through the metal through the holes mentioned above, and finally the metal vias (c_5_ and c_6_) on both sides of the resistance connection were grounded.

**Figure 8:**
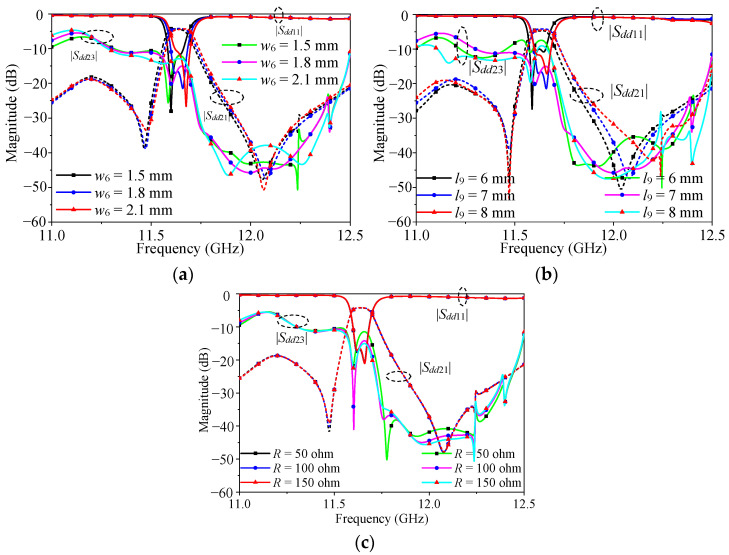
Effect of the isolation structure on power divider performance: (**a**) different *w*_6_; (**b**) different *l*_9_; (**c**) different *R*.

Figure 8 exhibits the performance of the proposed filtering power divider under different parameters of the isolation structure. It can be seen from Figure 8a,b that the length and width of the isolated metal wire had an impact on the performance of the power divider. By changing the size of these two parameters, an appropriate length and width of the metal wire could be obtained. Figure 8c shows the influence of different resistance values on the performance of the power divider. It was found that the resistance value only affected the isolation performance, so the appropriate resistance value could be selected according to this rule.

For the different desired operating frequency, the dimensions of the resonator should be chosen firstly, and then the feeding position and coupling gaps, as well as the isolation structure can be achieved based on the change rules in Figure 6 and Figure 8.

## 3. Results

**Figure 9:**
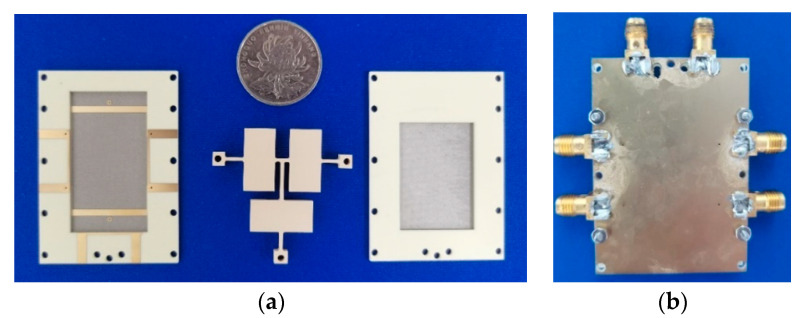
Photo of the proposed filtering power divider prototype: (**a**) photo of each part; (**b**) photo after assembling.

A prototype of the proposed dielectric resonator filtering power divider is demonstrated. Figure 9 shows a photograph of the simulated and measured results of the prototype, which were measured by the Keysight N5230C vector network analyzer. It can be seen from Figure 9 that this power divider was divided into three parts for processing. The dielectric block part, Substrates 1 and 2, and Substrates 3 and 4. Then, they were assembled and fixed with screws.

**Figure 10:**
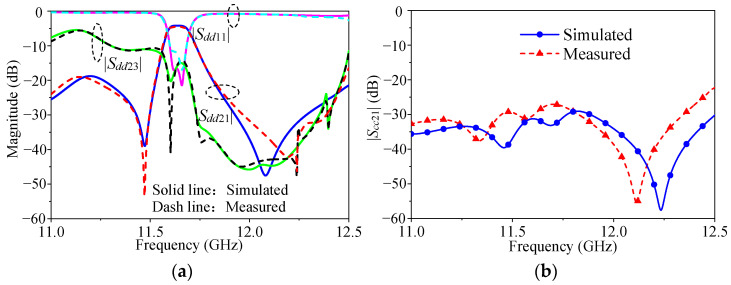
Simulated and measured results of the proposed filtering power divider prototype: (**a**) differential-mode response; (**b**) common-mode response.

Figure 10 indicates that the measured minimum differential-mode insertion loss was about 1.29 dB with a center frequency of 11.65 GHz, and the measured 3 dB FBW of 1.17% and the isolation could reach 15 dB within the operating band. The measured common-mode suppression was higher than 26.8 dB. The difference between the simulated and the measured results was caused by processing and equipment errors.

Table 1 lists the performance of this work and the state-of-the-art designs. Compared with reported metal resonator filtering power dividers [18,19,20,22], the proposed one was based on a dielectric resonator, which is more suitable for operating at high frequencies. Compared with reported balanced dielectric resonator filtering power dividers [23,24,25], the proposed design was realized based on a substrate and dielectric resonator and has the advantages of a low profile, as well as additional isolation between the output ports.

## 4. Conclusions

A low-profile balanced dielectric resonator filtering power divider is proposed. It was realized by using a grounded and hollow substrate combined with three coupled dielectric resonators. The ${TE}_{111}^{y}$ mode can be transmitted and divided under a differential-mode feed. By utilizing the clever hollow structure of its substrate stacking, isolation resistor structures were introduced at the two output ports to improve the isolation level of the power divider. The proposed design has both the features of a low profile and additional isolation between the output ports. Therefore, it is believed that the proposed balanced dielectric resonator filtering power divider is promising for modern wireless communication systems.

## Figures and Tables

**Table 1:** Comparison with reported balanced filtering power dividers.

Ref. No.	*f*_0_ (GHz)	3 dB FBW (%)	Insertion Loss (dB)	Isolation (dB)	Common-Mode Suppression (dB)	Profile (*λ*_g_)	Technology
[18]	2.76	15.9	0.6	18	45	N.A	Microstrip resonator
[19]	2.36	7	2.21	20	60	N.A	Microstrip resonator
[20]	1.8	11.7	1.02	32.1	55	N.A	Patch resonator
[22]	4.2/9.1	24/6	0.6/1.26	28/21	38/26	N.A	Patch resonator
[23]	1.52/1.64	0.84/0.94	0.9/0.85	4	N.A	2.78	Cavity + dielectric resonator
[24]	1.74	1.6	0.49	N.A	N.A	1.077	Cavity + dielectric resonator
[25]	3.49	1.95	0.92	N.A	43	1.04	Cavity + dielectric resonator
This work	11.65	1.17	1.29	15.1	26.8	0.66	Substrate + dielectric resonator

*λ*_g_: The guide wavelength at the center frequency.

## Data Availability

The data presented in this study are available on request from the corresponding author.
